# Atypical Presentation of Uterine Rupture in a Post-myomectomy Scar at 35+5 Weeks Gestation: A Case Report

**DOI:** 10.7759/cureus.96537

**Published:** 2025-11-11

**Authors:** Maysoon A Fahad, Anaum A Kabeer, Marim S Bakhiet, Sayed A Hassan

**Affiliations:** 1 Obstetrics and Gynecology, HMS (Health and Medical Services) Al Garhoud Hospital, Dubai, ARE; 2 Medicine, HMS (Health and Medical Services) Al Garhoud Hospital, Dubai, ARE; 3 Emergency Medicine, Father Muller Medical College Hospital, Mangalore, IND

**Keywords:** atypical presentation, breech delivery, emergency cesarean, fetal bradycardia, fundal rupture, hemoperitoneum, myomectomy scar, primigravida, third trimester pregnancy, uterine rupture

## Abstract

Uterine rupture in the third trimester is a rare but life-threatening obstetric emergency, particularly in women with a history of uterine surgery such as myomectomy. This report describes an atypical presentation in a 43-year-old primigravida with a prior fundal myomectomy. At 35+5 weeks of gestation, she presented to the emergency department with nonspecific abdominal discomfort, diarrhea, and persistent vomiting for over 8 hours. Notably, she had missed her 32-week antenatal visit and did not respond to calls made 2 days prior regarding elective cesarean scheduling, later citing fear of the hospital environment. Her antenatal care had otherwise been regular. The pregnancy was planned, and she conceived four months after a fundal myomectomy performed on 29 May 2024. She had been counseled regarding the associated risks but was keen to conceive early due to her age. Consequently, her pregnancy was classified as high risk owing to a fresh uterine scar.

On initial evaluation, the patient was hypotensive (BP 90/60 mmHg) and tachycardic (HR 100 bpm), and she reported perceiving normal fetal movements at that time. Fetal monitoring was initiated, and one hour into observation, cardiotocography revealed fetal bradycardia, with heart rates fluctuating between 60 and 130 bpm. An emergency lower-segment cesarean section under general anesthesia was performed. Intraoperatively, a massive hemoperitoneum and extensive intraperitoneal clots were discovered along with a full-thickness rupture at the fundal uterine scar. A breech fetus was delivered, the uterus was repaired in two layers, and one unit of packed red blood cells was transfused. The patient was monitored in the high-dependency unit and recovered well.

This case emphasizes the importance of maintaining a high index of suspicion for uterine rupture, even in the absence of classic signs such as severe abdominal pain or vaginal bleeding. Early fetal monitoring, timely diagnosis, and prompt surgical intervention are essential to improving maternal and fetal outcomes in women with prior uterine surgery.

## Introduction

Uterine rupture is a rare but potentially catastrophic event in obstetrics, with an incidence of approximately 0.07% in developed countries [1]. It is more commonly associated with prior cesarean deliveries; however, other uterine surgeries such as myomectomy, especially when involving the fundus, can also predispose to rupture [2]. Among women with a history of myomectomy, the reported incidence of uterine rupture ranges between 0.2% and 1%, depending on the depth and location of the incision. The underlying mechanism involves poor healing of the uterine muscle and weakness at the scar site, which can reduce the strength of the uterine wall during pregnancy, as commonly described in post-myomectomy healing. The risk increases significantly in the third trimester due to progressive uterine distension and wall thinning [3].

This case report describes an atypical presentation of uterine rupture in a primigravida with a history of fundal myomectomy. The patient presented at 35+5 weeks of gestation without the classical signs and symptoms of rupture, which contributed to a delay in immediate recognition. Given the growing number of women conceiving after uterine surgeries, this case underscores the importance of maintaining a high index of suspicion for uterine rupture, even in the absence of classical features.

## Case presentation

A 43-year-old primigravida at 35 weeks and 5 days of gestation presented to the emergency department on 28 May 2025. She complained of diarrhea and persistent vomiting for over eight hours, accompanied by nonspecific abdominal discomfort. Notably, she denied any significant abdominal pain or vaginal bleeding.

On examination, she was alert and oriented but showed early signs of hemodynamic instability with a blood pressure of 90/60 mmHg and a heart rate of 100 beats per minute. Her abdomen was soft with mild generalized tenderness. Fetal monitoring was initiated upon arrival.

Routine blood investigations were sent during the initial evaluation. The results revealed significant anemia, leukocytosis, elevated inflammatory markers, and mild metabolic acidosis. These findings are summarized in Table 1. Given her predominant gastrointestinal symptoms and blood investigations, the initial working diagnosis in the emergency department was suspected gastroenteritis.

**Table 1:** Abnormal laboratory findings at presentation Laboratory values at the time of admission showing anemia (low hemoglobin, hematocrit, and red blood cell count), leukocytosis with neutrophilia, elevated inflammatory markers (CRP), and mild metabolic acidosis. These findings suggest ongoing physiological stress and possible systemic inflammatory response related to uterine rupture and hemoperitoneum.

Parameter	Result	Normal Range	Interpretation
Hemoglobin	8.9 g/dL	12.5–15 g/dL	Low – Anemia
Hematocrit	27%	36–46%	Low – Consistent with anemia
RBC Count	3.1 ×10⁶/µL	3.8–4.8 ×10⁶/µL	Low
WBC Count	24.95 ×10³/µL	4–11 ×10³/µL	Elevated – Possible stress/infection
Neutrophils	22.31 ×10³/µL	2–7 ×10³/µL	Elevated
CRP	61.7 mg/L	<5.0 mg/L	Elevated – Inflammatory response
Bicarbonate	17 mmol/L	22–30 mmol/L	Low – Possible metabolic acidosis
Chloride	113 mmol/L	96–111 mmol/L	Slightly elevated
RDW	16%	11.6–14%	Elevated – Red cell variation

**Figure 1:**
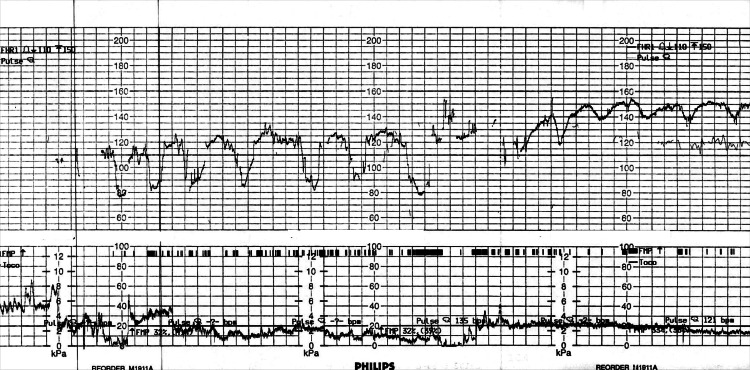
Cardiotocography showing fetal bradycardia Cardiotocography shows prolonged fetal bradycardia, with heart rate fluctuations between 60 and 130 bpm. This pattern of fetal distress prompted an emergency cesarean section. Note: baseline variability was reduced with absent accelerations and multiple decelerations.

Approximately one hour after presentation, cardiotocography (CTG) was performed. The CTG revealed fetal bradycardia with heart rates fluctuating between 60 and 130 beats per minute. These findings are illustrated in Figure 1.

**Figure 2:**
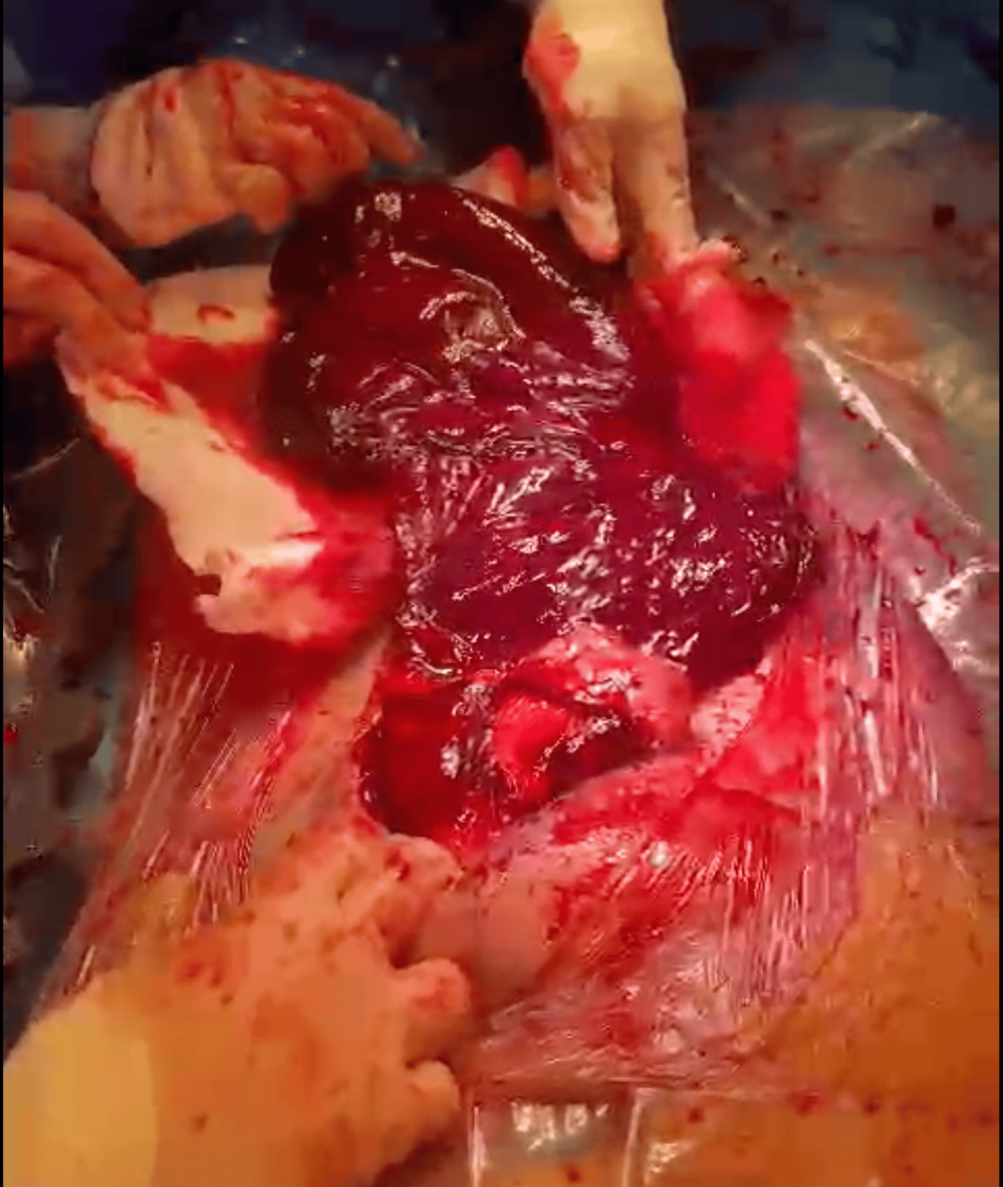
Intraoperative image showing massive hemoperitoneum and intraperitoneal clots Intraoperative photograph taken during the emergency cesarean section demonstrating a large volume of hemoperitoneum with extensive intraperitoneal blood clots, revealing a rupture at the fundal myomectomy scar.

Due to the fetal distress, the patient was admitted and prepared for an emergency cesarean section. Under general anesthesia, a transverse lower abdominal incision was performed. Upon entering the peritoneal cavity, a massive hemoperitoneum and extensive intraperitoneal clots were identified, as shown in Figure 2. A rupture was discovered in the fundal region of the uterus corresponding to the prior myomectomy scar. The fetus was delivered in breech presentation and transferred immediately to the neonatal team. The uterus was repaired in two layers, and hemostasis was achieved. Approximately 1.5 liters of blood loss was estimated, and 1 unit of packed red blood cells was transfused intraoperatively.

Postoperatively, the patient was transferred to the high-dependency unit for monitoring. She remained stable with adequate urine output and normal vital signs. Hemoglobin measured 10.2 g/dL after surgery. The patient recovered well and was subsequently moved to the general ward.

## Discussion

Uterine rupture is a rare but potentially life-threatening obstetric emergency, particularly in women with previous uterine surgery such as myomectomy [1]. The risk of rupture after myomectomy is influenced by factors including the location and depth of the uterine incision, the interval between surgery and conception, and the extent of uterine healing [2]. Fundal myomectomy scars are especially prone to rupture due to the relatively thinner myometrium in this region, which predisposes to dehiscence under the stress of advancing pregnancy [2,3].

In this case, the patient spontaneously conceived approximately four months after a fundal myomectomy, which is a shorter interval than typically recommended. Current guidelines generally advise delaying conception for at least 6 to 12 months post-myomectomy to allow adequate uterine healing and reduce the risk of rupture [4,5]. Early conception over a fresh scar increases the risk of poor myometrial remodeling and subsequent rupture during the third trimester.

The clinical presentation of uterine rupture is often dramatic, with severe abdominal pain, vaginal bleeding, and signs of maternal and fetal compromise [6]. However, this case illustrates an atypical presentation, where the patient exhibited nonspecific gastrointestinal symptoms without the classic triad. Similar reports have noted that uterine rupture may occasionally mimic gastrointestinal or urinary tract pathology, delaying diagnosis [7,8]. Such atypical symptoms highlight the need for heightened clinical suspicion in high-risk patients, even in the absence of hallmark features.

Fetal distress, as indicated by bradycardia and abnormal heart rate variability, remains one of the most sensitive indicators of uterine rupture [9]. In this case, CTG abnormalities prompted timely evaluation and expedited surgical intervention, underscoring the importance of vigilant fetal monitoring in patients with a history of uterine surgery.

Surgical management involved prompt cesarean delivery and uterine repair. While hysterectomy is sometimes unavoidable in cases of uncontrolled bleeding or extensive rupture, uterine preservation is prioritized in younger and primigravid women desiring future fertility [10]. Although maternal mortality from uterine rupture is low with timely intervention, potential operative complications include hemorrhage, need for blood transfusion, injury to adjacent organs, and, in rare cases, hysterectomy. Postoperative complications can include infection, adhesions, and impaired uterine integrity, which may affect future pregnancies. Early recognition and swift multidisciplinary action are essential to achieving favorable maternal and neonatal outcomes.

This case emphasizes the importance of comprehensive antenatal planning, adherence to recommended interpregnancy intervals after myomectomy, and patient education regarding risks associated with fresh uterine scars. Uterine repair generally allows for preservation of fertility, but patients should be counseled regarding the increased risk of recurrence in subsequent pregnancies. Careful preconception planning, close monitoring, and delivery by cesarean section in future pregnancies are recommended to minimize maternal and fetal risks. Furthermore, it demonstrates that clinicians should maintain a high index of suspicion for uterine rupture, even when patients present with atypical or nonspecific symptoms, to facilitate timely diagnosis and intervention.

## Conclusions

This case demonstrates that uterine rupture can present atypically, particularly in primigravida women with a history of uterine surgery such as myomectomy. In this patient, despite the absence of classic symptoms like severe abdominal pain or vaginal bleeding, laboratory abnormalities and fetal bradycardia were key indicators, emphasizing the importance of a high index of suspicion for timely diagnosis. Early recognition, fetal monitoring, and prompt surgical intervention were crucial in achieving favorable maternal and neonatal outcomes. While this case provides important lessons, it represents a single patient, and findings may not be generalizable. Adequate counseling regarding appropriate interpregnancy intervals after uterine surgery is essential, and women with recent myomectomy scars should be advised to report any unexplained abdominal or gastrointestinal symptoms promptly.

## References

[1] Kim HS, Oh SY, Choi SJ (2016). Uterine rupture in pregnancies following myomectomy: a multicenter case series. Obstet Gynecol Sci.

[2] Claeys J, Hellendoorn I, Hamerlynck T (2014). The risk of uterine rupture after myomectomy: a systematic review. Gynecol Surg.

[3] Parker WH, Einarsson J, Istre O, Dubuisson JB (2010). Risk factors for uterine rupture after laparoscopic myomectomy. J Minim Invasive Gynecol.

[4] Andersen MM, Thisted DL, Amer-Wåhlin I, Krebs L (2016). Can intrapartum cardiotocography predict uterine rupture among women with prior caesarean delivery?: a population based case-control study. PLoS One.

[5] Sugai S, Sasabuchi Y, Yasunaga H, Isogai T, Yoshihara K, Nishijima K (2024). In-hospital outcomes of repair and hysterectomy for uterine rupture: a nationwide observational study. Eur J Obstet Gynecol Reprod Biol.

[6] Yang H, Zhao Y, Tu J, Chang Y, Xiao C (2024). Clinical analysis of incomplete rupture of the uterus secondary to previous cesarean section. Open Med (Wars).

[7] TeachMeObGyn TeachMeObGyn (2024). TeachMeObGyn. Uterine rupture - risk factors, management, surgery. https://teachmeobgyn.com/labour/emergencies/uterine-rupture/.

[8] Cleveland Clinic (2022). Cleveland Clinic. Uterine rupture: signs, symptoms, risks & treatment. https://my.clevelandclinic.org/health/diseases/24480-uterine-rupture.

[9] Overtoom EM, Huynh TN, Rosman AN (2024). Predicting the risks and recognizing the signs: a two-year prospective population-based study on pregnant women with uterine rupture in The Netherlands. J Matern Fetal Neonatal Med.

[10] Wan S, Yang M, Pei J (2022). Pregnancy outcomes and associated factors for uterine rupture: an 8 years population-based retrospective study. BMC Pregnancy Childbirth.

